# A Kansas Reformer

**Published:** 1900-02

**Authors:** 


### TEXAS MEDICAL JOURNAL.

AUSTIN, TEXAS.

A MONTHLY JOURNAL OF MEDICINE AND SURGERY.

EDITED AND PUBLISHED BY

F. E. DANIEL, M. D., AND S. E. HUDSON, M. D.

Published Monthly at Austin, Texas, by Drs. Daniel and Hudson. Subscription price $1.00 a year in advance.

Eastern Representative: John Guy Monihan, St. Paul Building, 220 Broadway, New York City.

Official organ of the West Texas Medical Association, the Houston District Medical Association, the Austin District Medical Society, the Brazos Valley Medical Association, the Galveston County Medical Society, and several others.

**"WHAT WOULD JESUS DO?"**

A Kansas Reformer. The Journal has received a typewritten letter from the owners of the *Topeka Daily Capital,* a so-called "religious" paper, announcing with a great flourish, that "Rev. Charles M. Sheldon, the author of 'In His Steps' (presumably Christ's), will assume entire editorial and business control of the *Capital* on March 13th, prox., to illustrate his conception of what a Christian daily newspaper ought to be." This letter was soon followed by a copy of the *Topeka Daily Capital,* in order, I suppose, to give me some idea what a "Christian Daily Newspaper" is; that I might understand the nature and extent of the reform this theological Hercules is going to undertake; some idea of the nastiness in the "religious" Augean stable. The front page is adorned with a hideous wood cut of the reverend gentleman who is now in training for this Herculean feat, the belt and the championship of the world, which cut we were irreverent enough and unsophisticated enough to at first take for Jeffries, the prize fighter in another arena. Mr. Sheldon's labors will not begin till 13th March, and his *carte blanche* as to reforming is to last just six days. We will await with bated breath the first issue. We are led to expect a miracle inasmuch as the *Topeka Daily Capital* in making the announcement in the issue before us in box-car-letters, treats the subject editorially and says: "The influence with which it may be fraught for the the issue before us in box-car-letters, treats the subject editorially far as to repeat a fact which seems almost a miracle."

What fact? Look out, Brother Sheldon. Beware the fate of the reformer!

Of Mr. Sheldon's tract the *Topeka Daily Capital* says: "His spoken word to a handful found the printed page and became a moral lever second in power—reverently it may be said—to the Bible itself."

And adds: "Its policy (The *Topeka Daily Capital* under Mr. Sheldon) will be dictated by an interrogation point. 'What would Jesus do?' will be the challenge to every piece of news, to every editorial, to every advertisement."

"What would Jesus do?" (presumably if he were called upon to "illustrate his conception of a Christian daily newspaper") I give it up; I don't know what he would do. But I hazard the guess that He would, first thing, weed out the nasty, salacious, suggestive, indecent, lying, revolting and disgusting advertisements which I don't hesitate to say characterize the so-called religious press; more properly the church papers, and make them an abomination and a stench in the nostrils of all decent and self-respecting persons, Christian or heathen; unfit to go into the sacred precincts of any man's home. As Mr. Sheldon is to follow "In His Steps" we suggest that as "charity begins at home" so should moral sanitation, and he can't do better than begin his reform with the *Topeka Daily Capital.* Lest he should be too much taken up with the "far reaching effects" of his tract, which, the *Topeka Daily Christian Capital* says is second only to the Bible, I venture to put my finger on some advertisements that are not calculated, in my judgment, to inspire holy thoughts and pure lives. For instance, passing over the familiar "Lost Manhood" and "Impotence" ads., with their suggestive and lying statements adorned with a hideous picture of some old quack, —they are too familiar—they are in all the papers, we point out a few that are supposed to appeal especially to the Y. M. C. A. young men, and the "sisters" of the Christian families where the *Topeka Daily Capital* circulates. The boys who have been guilty of "youthful indiscretions" are told that big "G" cures gonorrhœa, which these Christian young men are, by implication at least, supposed to have. The good Christian ladies who are thinking of their "busts" are told how they can "get them expanded six inches;" and "Ladies" who wish to get dilated are requested to ask their druggists to "show them their Daisy Dilators" (whatever they may be); while, should any good Christian lady find herself dilated or dilating she is kindly informed by her religious paper where the best pennyroyal pills may be had.

